# Routine versus on demand removal of the syndesmotic screw; a protocol for an international randomised controlled trial (RODEO-trial)

**DOI:** 10.1186/s12891-018-1946-5

**Published:** 2018-01-31

**Authors:** S. A. Dingemans, M. F. N. Birnie, F. R. K. Sanders, M. P. J. van den Bekerom, M. Backes, E. van Beeck, F. W. Bloemers, B. van Dijkman, E. Flikweert, D. Haverkamp, H. R. Holtslag, J. M. Hoogendoorn, P. Joosse, M. Parkkinen, G. Roukema, N. Sosef, B. A. Twigt, R. N. van Veen, A. H. van der Veen, J. Vermeulen, J. Winkelhagen, B. C. van der Zwaard, S. van Dieren, J. C. Goslings, T. Schepers

**Affiliations:** 1 Department of Surgery, Trauma Unit, Academic Medical Centre, University of Amsterdam, P.O. Box 22660, 1100 DD Amsterdam, The Netherlands; 2 Department of Orthopedic Surgery, OLVG, P.O. Box 95500, 1090 HM Amsterdam, The Netherlands; 3 Department of Public Health, Erasmus MC, P.O. Box 2040, 3000 CA Rotterdam, The Netherlands; 4 Department of Surgery, Trauma Unit, VU University Medical Centre, P.O. Box 7057, 1007 MB Amsterdam, The Netherlands; 5 Department of Surgery, Flevo Hospital, P.O. Box 3005, 1300 EG Almere, The Netherlands; 6 Department of Surgery, Deventer Hospital, P.O. Box 5001, 7400 GC Deventer, The Netherlands; 7 Department of Surgery, Slotervaart Hospital, P.O. Box 90440, 1006BK Amsterdam, The Netherlands; 8 Department of Surgery, Haaglanden MC, P.O. Box 432, 2501 CK The Hague, The Netherlands; 9 Department of Surgery, Noordwest Hospital Group, P.O. Box 501, 1815 JD Alkmaar, The Netherlands; 10 Department of Orthopaedics and Traumatology, Helsinki University Hospital, Topeliuksenkatu 5, 00260 Helsinki, Finland; 11 Department of Surgery, Maasstad Hospital, P.O. Box 9100, 3007 AC Rotterdam, The Netherlands; 12 Department of Surgery, Spaarne Hospital, P.O. Box 770, 2130 AT Hoofddorp, The Netherlands; 13 Department of Surgery, BovenIJ Hospital, P.O. Box 37610, 1030 BD Amsterdam, The Netherlands; 14 Department of Surgery, OLVG, P.O. Box 95500, 1090 HM Amsterdam, The Netherlands; 15 Department of Surgery, Catharina Hospital, P.O. Box 1350, 5602 ZA Eindhoven, The Netherlands; 16 Department of Surgery, Westfries Hospital, P.O. Box 600, 1620 AR Hoorn, The Netherlands; 17 Department of Orthopaedics, Jeroen Bosch Hospital, P.O. Box 90153, 5200 ME ‘s-Hertogenbosch, The Netherlands

**Keywords:** Syndesmosis, Syndesmotic screw, Routine removal, Removal on demand, Functional outcome

## Abstract

**Background:**

Syndesmotic injuries are common and their incidence is rising. In case of surgical fixation of the syndesmosis a metal syndesmotic screw is used most often. It is however unclear whether this screw needs to be removed routinely after the syndesmosis has healed. Traditionally the screw is removed after six to 12 weeks as it is thought to hamper ankle functional and to be a source of pain. Some studies however suggest this is only the case in a minority of patients. We therefore aim to investigate the effect of retaining the syndesmotic screw on functional outcome.

**Design:**

This is a pragmatic international multicentre randomised controlled trial in patients with an acute syndesmotic injury for which a metallic syndesmotic screw was placed. Patients will be randomised to either routine removal of the syndesmotic screw or removal on demand. Primary outcome is functional recovery at 12 months measured with the Olerud-Molander Score. Secondary outcomes are quality of life, pain and costs. In total 194 patients will be needed to demonstrate non-inferiority between the two interventions at 80% power and a significance level of 0.025 including 15% loss to follow-up.

**Discussion:**

If removal on demand of the syndesmotic screw is non-inferior to routine removal in terms of functional outcome, this will offer a strong argument to adopt this as standard practice of care. This means that patients will not have to undergo a secondary procedure, leading to less complications and subsequent lower costs.

**Trial registration:**

This study was registered at the Netherlands Trial Register (NTR5965), Clinicaltrials.gov (NCT02896998) on July 15th 2016.

## Background

Ankle fractures are among the most common fractures. It is estimated that the incidence of ankle fractures ranges from about 25,000 in the Netherlands to more than five million people in the United States annually and the incidence is rising [1, 2]. Both young and elderly people are at risk for these fractures. In general, younger people are more at risk as a result of a more active lifestyle and elderly people because of poorer bone quality [3, 4]. Approximately half of the patients with an ankle fracture require surgical treatment because of joint instability. In approximately 20% of these fractures there is a concomitant injury of the syndesmosis and syndesmotic repair is indicated [5]. After anatomical reduction a syndesmotic ‘positioning screw’ is placed through the fibula into the tibia to maintain this reduction and allow the syndesmotic ligaments to heal. Extensive research has been conducted regarding the technical aspects of the placement of the syndesmotic screw. For example, the number of screws, their diameter, the level of placement and whether they should engage three or four cortices have been investigated thoroughly [6–10]. After a period of 8 – 10 weeks the syndesmosis will generally be healed and the screw will lose its function. It is an ongoing discussion whether the syndesmotic screw needs to be removed subsequently. Most surgeons advocate its removal because of suspected impaired range of motion and chance of breakage of the screw [9, 11–13]. During normal ambulation the fibula moves and the syndesmosis widens [14, 15]. The positioning screw is thought to restrict this movement and the screw is therefore removed. However, several case series have shown similar outcomes in patients in which the syndesmotic screw was retained compared to patients in whom the syndesmotic screw was removed [16–18]. The positioning screw is most likely not causing complaints in patients with retained screws because of loosening or breakage of the screw [16, 17, 19, 20]. In a recent systematic review there seemed to be no significant difference in functional outcome between patients undergoing routine removal and patients in whom the syndesmotic screw was only removed in case of symptomatic implants. [21] However, this was only based on one underpowered RCT and several low quality case-series and therefore high-quality evidence on this subject is desirable.

We therefore initiated a randomized controlled trial in which we aim to investigate the effect of ‘removal of demand’ of the syndesmotic screw(s) compared to ‘routine removal’ on functional outcome. Furthermore we will investigate the economic effect of leaving the syndesmotic screw(s) in place.

### Design

This pragmatic international multicentre randomised controlled trial will randomise between routine- and on demand removal of the syndesmotic screw(s) after placement for a traumatic syndesmotic injury (both isolated syndesmotic injuries and concomitant syndesmotic injuries in ankle fractures). Both teaching and non-teaching hospitals will participate in this study including three academic Level 1 trauma centres in Europe. An overview of participating centres is shown in Table 1.

**Table 1:** Participating centres

Academic Medical Centre^a^	Amsterdam, the Netherlands
Bovenij Hospital	Amsterdam, the Netherlands
Catharina Hospital	Eindhoven, the Netherlands
Deventer Hospital	Deventer, the Netherlands
Flevo Hospital	Almere, the Netherlands
Haaglanden MC	The Hague, the Netherlands
Helsinki University Hospital^a^	Helsinki, Finland
Jeroen Bosch Hospital	‘s-Hertogenbosch, the Netherlands
Maasstad Hospital	Rotterdam, the Netherlands
Noordwest Hospital Group	Alkmaar, the Netherlands
OLVG	Amsterdam, the Netherlands
Slotervaart Hospital	Amsterdam, the Netherlands
Spaarne Hospital	Amsterdam, the Netherlands
VU University Medical Centre^a^	Amsterdam, the Netherlands
Westfries Hospital	Hoorn, the Netherlands

^a^level 1 trauma centres

### Participants

The eligible study population will consist of consecutive adult patients with a traumatic syndesmotic injury.

### Inclusion criteria

- Placement of one or two metallic syndesmotic screw(s) for an unstable ankle fracture with a syndesmotic injury or an isolated syndesmotic injury
- Syndesmotic screw(s) placed within 2 weeks of the trauma
- Physical condition allows the patient to undergo an elective second procedure (i.e. removal of the screw)

### Exclusion criteria

- ISS score > 15
- Injuries to the ipsi- and contralateral side which may hamper rehabilitation
- Other medical conditions which hamper physical rehabilitation (i.e. musculoskeletal disabilities or severe psychological conditions)
- Insufficient understanding of the Dutch or English language

### Interventions

Patients will be informed about the study by their treating physician following the procedure in which the syndesmotic screw was placed. After this, patients are contacted by the coordinating investigator to request participation in the study. After obtaining signed informed consent patients will be randomly assigned in a 1:1 allocation ratio to one of the following groups:

1. Control group: Routine removal of the syndesmotic screw(s) 8 – 12 weeks following the index procedure

or

2) Intervention group: On demand removal of the syndesmotic screw(s)

Patients in the control group will undergo routine removal of the syndesmotic screw 8 – 12 weeks post-operatively (according to the preference of the treating surgeon). Patients will not undergo routine removal in case of 1) a contra-indication for undergoing a second procedure for example due to a (new) medical condition or 2) explicit request of the patient after consultation of their treating surgeon. In the intervention group the screw will only be removed in case of symptomatic implants, defined as: 1) implants causing pain, 2) implants (suspected of) causing restricted range-of-motion 3) explicit request of the patient 4) an infection or 5) other problems related to the screw such as protruding screw-head. The screw will only be removed after a consultation of the treating surgeon (except in patients who wish to no longer participate in the study). In patients in the control group in whom the syndesmotic screw brakes prior to planned removal, the screw will be left in place and only removed in case of symptoms.

### Study procedures

This study is a pragmatic trial, which implies physicians are allowed to follow local guidelines concerning the treatment of these injuries apart from the intervention investigated. Participating centres are however informed that the preferred surgical technique is a tricortical 3.5 mm diameter screw between 2 and 4 cm of the pilon. If a large reduction clamp is used, the preferred technique is the use of a temporary K-wire as ‘glide path’ [22]. Besides this, participating centres are allowed to choose their own postoperative treatment routine: for example in the use of a cast, non-weight bearing regime and timing of syndesmotic screw removal (within the predefined time window). At 3 months following the index procedure, patients are assessed at the outpatient clinic. Patients are instructed to visit the outpatient clinic sooner in case of any signs of a POWI: warmth, redness, pain, drainage or a fever above 38.5 degrees Celsius. During the visit to the outpatient clinic the patients are seen by their treating physician and the coordinating investigator. The coordinating investigator will document signs of POWI and will determine its presence or any special findings on physical examination. Furthermore patients are requested to fill out several questionnaires (Table 1). At the six and 12 months follow-up, patients are requested to fill out the same questionnaires and the range-of-motion is measured. Follow-up will take place within a window of 2 weeks of the projected follow-up moment.

### Randomisation

Randomisation will be stratified by centre and age category (i.e. ≥ 60 years and < 60 years). Randomisation will be blocked within strata. Randomisation sequence is generated by a dedicated computer randomisation software program (CASTOR®, Amsterdam, The Netherlands), ensuring allocation concealment. Randomisation will mostly be performed at the outpatient clinic by coordinating investigator using a dedicated, password protected, SSL–encrypted website.

### Primary Outcome

The primary outcome parameter is functional outcome 12 months following the index procedure measured with the Olerud-Molander ankle score (OMAS) (Table 2).

**Table 2:** Time table and follow-up schedule

RODEO-trial	Enrollment	Randomization / Allocation	Follow-up		
TIMEPOINT	Post-operatively	8 – 12 weeks post-operatively	3 months Post-operatively	6 months Post-operatively	12 months Post-operatively
Enrollment					
Eligibility screening	X				
Informed Consent	X				
Intervention					
Removal of syndesmotic screw (according to randomization)		X			
Assessment					
Plain radiographs	X		X		
OMAS			X	X	X
Visual analogue pain scale (VAS)			X	X	X
Range-of-Motion			X	X	X
POWI			X		
AOFAS			X	X	X
EQ-5D-5 L			X	X	X
i-MCQ			X	X	X
i-PCQ			X	X	X

To be able to assess the Minimally Clinical Important Difference of the OMAS, anchor questions will be added to the OMAS at six and 12 months as described by Walenkamp et al. [23].

### Secondary outcomes

#### Secondary outcome measures of the study are:

- Functional outcome with the American Orthopedic Foot and Ankle Hindfoot Score (AOFAS) [24]
- Pain as measured by a ten-point Visual Analog Scale.
- Range of motion, both absolute and as a percentage compared with the uninjured side.
- Postoperative wound infections classified using the criteria as defined by The Centers for Disease Control and Prevention (CDC-criteria) [25]
- Synostosis or recurrent diastasis (as seen on radiographs made in case of symptoms)
- Health-related quality of life as measured by the EQ-5D-5 L questionnaire [26].
- Health care resources utilization (including amongst others; number of visits to the general practitioner and use of home care organizations) as measured by way of a combination of the Dutch/English iMTA Medical Consumption Questionnaire (iMCQ) and iMTA Productivity Cost Questionnaire (iPCQ) (only applicable for the Dutch study population).
- Costs (economic evaluation including budget impact analysis): the economic evaluation of the RODEO-trial will be performed as a cost-effectiveness (CEA) as well as a cost-utility (CUA) analysis (only applicable for the Dutch study population).

Furthermore general demographics will be assessed such as age, gender, body mass index, co-morbidities, American Society of Anesthesiologists (ASA) classification, substance abuse, level of activity, bone mineral density (when available), fracture characteristics, surgical characteristics, duration of non-weight bearing period and use of physiotherapy.

### Sample size

We based our sample size calculation on a non-inferiority design. The Olerud-Molander score (OMAS) will serve as primary outcome measure. We have used the results from an earlier study on this subject for our sample size calculation [27]. For the sample size calculation we hypothesized an equal OMAS between the two groups. Using a one-sided significance level (α) of 0.025 and a power (ß) of 90% with a standard deviation (SD) of 19 points (derived from the study mentioned before) and setting our non-inferiority limit at 10 a total of 76 patients are needed in each study arm. Taking a 10% loss to follow-up into account, a total number of 167 subjects will be needed to demonstrate non-inferiority between the two treatment strategies. Furthermore we performed a sample size calculation for a subgroup analysis based on age. We hypothesize that the SD will be lower in these subgroups due to increased homogeneity, therefore we have used an SD of 16 for the sample size calculation of the subgroups. Using a significance level (α) of 0.05 and a power (ß) of 90% 88 patients are needed in each subgroup to prove non-inferiority. Taking 10% loss to follow-up into account a total of 193 patients need to be randomized.

### Statistical analysis

The primary endpoint will be analysed according to the intention-to-treat and the per-protocol principle, non-inferiority will only be declared if both types of analysis prove non-inferiority. The primary endpoint will be analysed using a one sided test for non-inferiority with an alpha of 0.025. Descriptive methods will be used to assess quality of data, homogeneity of treatment groups and endpoints. Normality of the data will be analysed by visually inspecting the histograms. Secondary outcomes will be analysed using either a t-test or Mann-Whitney U test for continuous data according to the distributing of the data and a Chi Square test for categorical data. Missing data will be handled through multiple imputation with predictive mean matching. All analyses will be performed using the standard statistical software. Separate analyses will be performed on subgroups based on age. A multivariable analysis will be performed to identify predictors of worse functional outcome.

The CEA and CUA will be performed on the intention to treat data, with a time horizon of 12 months and from a societal perspective. The primary economic outcomes are the costs per quality adjusted life year (QALY) and the costs per point functional recovery improvement. Moreover a budget impact analysis (BIA) will be performed with a time horizon of 4 years. The questionnaires estimating the secondary outcome measures ‘resources utilization’ and ‘costs’ will only be used in patients included in the Netherlands due to practical feasibility and to ensure a valid outcome.

### Recruitment and consent

The patient will be informed about the RODEO-trial following placement of a syndesmotic screw or when he or she visits the outpatient clinic following surgery and is provided with the patient information letter. Patients will have a minimum of 2 days to decide whether they want to participate or not in the study. For patients recruited directly postoperatively this means they can be included upon their first visit at the outpatient clinic. For patients who are informed for the first time at the outpatient clinic the coordinating investigator will contact them by phone (if the patient agrees to be contacted by phone by the coordinating investigator). Randomisation will take place after they have returned the signed informed consent forms.

### Benefits and risks assessment, group relatedness

A recent systematic review suggests that our intervention is safe and has similar functional outcome compared to the routine removal [21]. Subjects will not undergo additional investigations and interventions due to participation in the RODEO-trial and therefore risks to subjects involved in this trial are at least similar to current practice. Potential benefits for subjects in the investigational treatment arm could be a lower risk of surgical site infections and not having to undergo a secondary procedure.

### Indemnities

The institutional review board at the AMC has waived liability insurance, because no additional risk can be attributed to participation in this study.

### Publication plan

The principal investigator, the study designer and the study coordinator will be named author. All others will obtain group authorship in the study group. All authors including group members are allowed to present the results after approval of the principle investigator.

## Discussion

Displayed above is the protocol for an adequately powered study investigating the difference in routine removal versus removal on demand of the syndesmotic screw in ankle injuries. This will be the first RCT able to prove whether a statistically significant and clinically relevant difference exists.

Since this is a pragmatic trial, surgeons are allowed to choose their own postoperative treatment routine. This, combined with the 15 participating centres will result in a variation in for example the use of a cast, the duration of non-weight bearing mobilisation and a minor variation in the timing of the removal of the syndesmotic screw. However, we believe that this situation accurately reflects daily practice, considering that slight variations in post-operative treatment regimens are inevitable.

The inclusion of the University Hospital Helsinki makes this trial international. This greatly improves the external validity of the trial. Not all secondary outcome measures can be used in an international setting. The budget impact analysis and the health care resources utilization for example can only be used for patients in the Netherlands. This is due to practical feasibility but also to ensure a valid outcome. When the same (translated) questionnaire would be used in patients in Finland, results would not be extractable since the costs of healthcare (e.g. a surgical procedure or a visit to the physiotherapist) will not likely be the same as in the Netherlands. However, the participation of a hospital outside of the Netherlands will give us more insight in how to implement the results not just in the Netherlands, but in the rest of Europe as well.

If this trial proves that removal on demand is indeed non-inferior to routine removal of the syndesmotic screw(s) in terms of functional outcome, this will offer a strong argument to adopt this as standard practice of care. It would mean that patients will not have to undergo a secondary procedure, leading to less complications and subsequent lower costs.

## Abbreviations

CEA: Cost-effectiveness analysis
CUA: Cost-utility analysis
EQ-5D: EuroQuality of Life-5D
iMCQ: iMTA medical consumption questionnaire
iPCQ: MTA productivity cost questionnaire
N: Number
NA: Not available
POWI: Postoperative wound infection
QALY: Quality adjusted life year
RCT: Randomised controlled trial
