# Correction to: Routine versus on demand removal of the syndesmotic screw; a protocol for an international randomised controlled trial (RODEO-trial)

**DOI:** 10.1186/s12891-020-03516-7

**Published:** 2020-08-05

**Authors:** S. A. Dingemans, M. F. N. Birnie, F. R. K. Sanders, M. P. J. van den Bekerom, M. Backes, E. van Beeck, F. W. Bloemers, B. van Dijkman, E. Flikweert, D. Haverkamp, H. R. Holtslag, J. M. Hoogendoorn, P. Joosse, M. Parkkinen, G. Roukema, N. Sosef, B. A. Twigt, R. N. van Veen, A. H. van der Veen, J. Vermeulen, J. Winkelhagen, B. C. van der Zwaard, S. van Dieren, J. C. Goslings, T. Schepers

**Affiliations:** 1 Department of Surgery, Trauma Unit, Academic Medical Centre, University of Amsterdam, P.O. Box 22660, 1100 DD Amsterdam, The Netherlands; 2 Department of Orthopedic Surgery, OLVG, P.O. Box 95500, 1090 HM Amsterdam, The Netherlands; 3 Department of Public Health, Erasmus MC, P.O. Box 2040, 3000 CA Rotterdam, The Netherlands; 4 Department of Surgery, Trauma Unit, VU University Medical Centre, P.O. Box 7057, 1007 MB Amsterdam, The Netherlands; 5 Department of Surgery, Flevo Hospital, P.O. Box 3005, 1300 EG Almere, The Netherlands; 6 Department of Surgery, Deventer Hospital, P.O. Box 5001, 7400 GC Deventer, The Netherlands; 7 Department of Surgery, Slotervaart Hospital, P.O. Box 90440, 1006 BK Amsterdam, The Netherlands; 8 Department of Surgery, Haaglanden MC, P.O. Box 432, 2501 CK The Hague, The Netherlands; 9 Department of Surgery, Noordwest Hospital Group, P.O. Box 501, 1815 JD Alkmaar, The Netherlands; 10 Department of Orthopaedics and Traumatology, Helsinki University Hospital, Topeliuksenkatu 5, 00260 Helsinki, Finland; 11 Department of Surgery, Maasstad Hospital, P.O. Box 9100, 3007 AC Rotterdam, The Netherlands; 12 Department of Surgery, Spaarne Hospital, P.O. Box 770, 2130 AT Hoofddorp, The Netherlands; 13 Department of Surgery, BovenIJ Hospital, P.O. Box 37610, 1030 BD Amsterdam, The Netherlands; 14 Department of Surgery, OLVG, P.O. Box 95500, 1090 HM Amsterdam, The Netherlands; 15 Department of Surgery, Catharina Hospital, P.O. Box 1350, 5602 ZA Eindhoven, The Netherlands; 16 Department of Surgery, Westfries Hospital, P.O. Box 600, 1620 AR Hoorn, The Netherlands; 17 Department of Orthopaedics, Jeroen Bosch Hospital, P.O. Box 90153, 5200 ME ‘s-Hertogenbosch, The Netherlands

**Correction to: BMC Musculoskelet Disord 19, 35 (2018)**

**https://doi.org/10.1186/s12891-018-1946-5**

In the originally published version of this protocol [1], the sample size calculation of the subgroups for age should have been phrased differently. The actual sample size of each subgroup (routine removal ≥60 years old, routine removal < 60 years old, on demand removal ≥60 years old and on demand removal < 60 years old) should be 44, making a total of 176 (unchanged), which would come to a required number of included patients of 196 when incorporating a loss to follow up of 10%.
